# Ejaculate sperm number compensation in stalk-eyed flies carrying a selfish meiotic drive element

**DOI:** 10.1038/s41437-018-0166-y

**Published:** 2018-11-22

**Authors:** Lara C. Meade, Deidre Dinneen, Ridhima Kad, Dominic M. Lynch, Kevin Fowler, Andrew Pomiankowski

**Affiliations:** 1 Department of Genetics, Evolution and Environment, University College London, Gower Street, London, WC1E 6BT UK; 2 CoMPLEX, University College London, Gower Street, London, WC1E 6BT UK

**Keywords:** Evolutionary genetics, Sexual selection, Evolutionary biology, Genotype

## Abstract

Meiotic drive genes cause the degeneration of non-carrier sperm to bias transmission in their favour. Males carrying meiotic drive are expected to suffer reduced fertility due to the loss of sperm and associated harmful side-effects of the mechanisms causing segregation distortion. However, sexual selection should promote adaptive compensation to overcome these deleterious effects. We investigate this using SR, an X-linked meiotic drive system in the stalk-eyed fly, *Teleopsis dalmanni*. Despite sperm destruction caused by drive, we find no evidence that SR males transfer fewer sperm to the female’s spermathecae (long-term storage organs). Likewise, migration from the spermathecae to the ventral receptacle for fertilisation is similar for SR and wildtype male sperm, both over short and long time-frames. In addition, sperm number in storage is similar even after males have mated multiple times. Our study challenges conventional assumptions about the deleterious effects of drive on male fertility. This suggests that SR male ejaculate investment per ejaculate has been adjusted to match sperm delivery by wildtype males. We interpret these results in the light of recent theoretical models that predict how ejaculate strategies evolve when males vary in the resources allocated to reproduction or in sperm fertility. Adaptive compensation is likely in species where meiotic drive has persisted over many generations and predicts a higher stable frequency of drive maintained in wild populations. Future research must determine exactly how drive males compensate for failed spermatogenesis, and how such compensation may trade-off with investment in other fitness traits.

## Introduction

Meiotic drive genes are segregation distorters which manipulate the products of gametogenesis so as to bias transmission in their favour (Burt and Trivers 2006; Lindholm et al. 2016). They have been observed in a diverse range of taxa, including plants, fungi, mammals and insects (Taylor 1999; Jaenike 2001; Burt and Trivers 2006). Sex-ratio (SR) meiotic drive occurs when the driver is located on the X-chromosome and acts against Y-bearing gametes (or vice versa), producing distorted brood sex ratios. Both autosomal and sex chromosome meiotic drive are common in males (Taylor and Ingvarsson 2003) where they are expected to cause sperm limitation, as abnormal sperm development has been widely reported in drive males (Novitski et al. 1965; Tokuyasu et al. 1977; Lyttle 1991; Cobbs et al. 1991; Wood and Newton 1991; Presgraves et al. 1997; Cazemajor et al. 2000; Wilkinson and Sanchez 2001; Keais et al. 2017). This deficit could slow the spread and lower the equilibrium frequency of drive if drive males are unable to deliver as many sperm per copulation or mate as often as wildtype males, and may suffer disproportionately when there is sperm competition (Taylor and Jaenike 2002; Holman et al. 2015).

Here we investigate whether alterations to ejaculate allocation might allow drive males to cope better with the detrimental effect of meiotic drive. This is explored by measuring the amount of sperm stored by females mated to drive and wildtype males. Male ejaculate allocation strategies are predicted to evolve in response to the average degree of sperm competition faced by an ejaculate (Parker 1990, 1998; Wedell et al. 2002). Models that examine the co-evolution of male ejaculate expenditure with sperm competition, predict that males with continuously varying resource levels are generally not expected to vary their levels of investment per ejaculate (Tazzyman et al. 2009). If males do not know how often individual females have mated previously or will mate in the future, they allocate their ejaculate given the expected level of sperm competition, which is independent of their own resources. Males with greater resources simply invest in a larger number of copulations. From this perspective, as drive males produce a larger fraction of non-functional sperm, they can be viewed as males with fewer resources to allocate to reproduction. Their limitation of resources leads to the prediction that drive males will make the same allocation per ejaculate as wildtype males, but simply reduce their number of matings. A related analysis was carried out by Engqvist (2012) who considered the allocation strategies of fertile and sub-fertile males. Under conditions of high sperm competition, this model predicts that sub-fertile males invest more per ejaculate than males with standard fertility (investment in addition varies with the frequency of the two male types). Drive males are sub-fertile in the sense that a proportion of the sperm they produce are non-functional and their drive-carrying sperm may be damaged as a by-product of the action of drive (Newton et al. 1976; Nasuda et al. 1998; Price and Wedell 2008). In both theoretical cases, there is no longer a straightforward expectation that drive males should deliver smaller ejaculates or have lower fertility per copulation.

We test these ideas which suggest that males do not alter ejaculate investment with variation in resources or fertility using the Malaysian stalk-eyed fly species *Teleopsis dalmanni*. Male carriers of X^SR^ (SR males), an X-linked meiotic drive system, produce strongly female-biased broods due to the failed maturation of Y-bearing sperm during spermatogenesis (Presgraves et al. 1997; Wilkinson and Sanchez 2001). In the extreme, one-half of sperm produced are non-functional (i.e., all Y-bearing sperm) leading to female only broods (Cotton et al. 2014; Paczolt et al. 2017). Populations of *T. dalmanni* have stable frequencies of X^SR^ (~10–30%), that have persisted across many generations (Wilkinson et al. 2003; Cotton et al. 2014), and drive is also found in its sister species *T. whitei* (Presgraves et al. 1997; Baker et al. 2001). This suggests that there has been ample time for selection to have driven adaptation of sperm allocation in drive males, which is consistent with experimental evolution in a variety of species showing that sperm allocation is a rapidly evolving trait (Ingleby et al. 2010; Firman and Simmons 2011; McNamara and Simmons 2017). Male *T. dalmanni* transfer few sperm per copulation (~65, Wilkinson et al. 2005; ~142, Rogers et al. 2006) as they partition their ejaculate across many matings, a strategy to cope with females that mate repeatedly with multiple males (Wilkinson et al. 1998). Females in the laboratory readily mate multiple times per day with a lifespan which can extend to >6 months (Reguera et al. 2004). Furthermore, there is no clear advantage to males in mating first or second in this species (Corley et al. 2006) and so total numbers of sperm may have been indicative of a male’s sperm competitive ability, as is assumed in sperm competition models based on a fair raffle (Parker 1998; Wedell et al. 2002; Tazzyman et al. 2009; Engqvist 2012). Consequently, SR male ejaculate investment is likely to have been selected in response to high levels of multiple mating and resulting sperm competition.

During copulation, male *T. dalmanni* attach a spermatophore, containing sperm and seminal fluid, to the base of the spermathecal ducts within the female reproductive tract (Kotrba 1996). Sperm are then transferred and stored in three sclerotised sacs (a singlet and doublet) that make up the spermathecae (Kotrba 1995; Presgraves et al. 1999). These are long-term sperm storage organs, and female *T. dalmanni* continue to lay fertilised eggs for around three weeks after a single mating (Rogers et al. 2006). To be used in fertilisation, sperm must move from the spermathecae to the ventral receptacle (VR) (Kotrba 1993; Rose et al. 2014). The gonopore of an egg lines up with the entrance of the VR as the egg passes through the female reproductive tract. The VR has multiple pouches, each of which can store a single sperm, and the proportion of sperm stored in the VR is predictive of male fertilisation success (Rose et al. 2014).

Measurements of spermatophore size and sperm number are not suitable to calibrate SR male investment per ejaculate. Spermatophore size in *T. dalmanni* can be measured (Rogers et al. 2006), but the structure is very small and compact (Kotrba 1996). The individual sperm are entangled, and numbers are impossible to quantify, even in the much larger spermatophores of the related stalk-eyed fly *D. meigenii* (Harley et al. 2013). We instead examined the number of sperm stored in the spermathecae. These organs can be dissected from the female and crushed to release sperm, which can then be accurately counted. In SR males, around 50% of sperm bundles in the testes can be visualised as degenerate (Presgraves et al. 1997). These non-viable Y-bearing sperm are not passed to the female and do not enter the spermathecae, as has been demonstrated in the related *T. whitei* (Fry and Wilkinson 2004). For a male to successfully achieve fertilisation, sperm must be able to survive in the spermathecae (i.e., long-term storage) and migrate to the VR. To gauge this, we examined the number of VR pouches that were filled at ~6 and at ~54 h after a single mating with an SR or a wildtype (ST) male.

Males may adopt ejaculate allocation strategies depending on female quality (Wedell et al. 2002; Reinhold et al. 2002; Harley et al. 2013). The female quality that most obviously affects male fitness is fecundity. In *T. dalmanni* fecundity is positively correlated with female eyespan (Rogers et al. 2006), and males prefer to mate with large eyespan females (Cotton et al. 2015) and give them larger spermatophores (Rogers et al. 2006). We used variation in female eyespan as an indicator of fecundity, and counted the sperm in the spermathecae as a measure of sperm allocation. We expected males to allocate a greater quantity of sperm per copulation to large females, as has been found in the related stalk-eyed fly *D. meigenii* (Harley et al. 2013), and tested whether this was also the case in SR males. In a final pair of experiments, males were mated sequentially to three females and we examined sperm numbers in the spermathecae, to test whether SR males become sperm depleted sooner than ST males.

## Methods

### Stock source and maintenance

Flies for the standard stock (ST-stock) population were collected (by S. Cotton and A. Pomiankowski) in 2005 from the Ulu Gombak valley, Peninsular Malaysia (3°19′N 101°45′E). Subsequently, flies were maintained in high density cages (> 200 individuals) to minimise inbreeding. This stock carries the wildtype X chromosome (X^ST^). It has been regularly monitored and does not contain meiotic drive.

Flies for the meiotic drive stock (SR-stock) population contain a sex-ratio distorting X chromosome (X^SR^). They were collected from the same location in 2012 (by A. Cotton and S. Cotton). To establish and maintain a stock with meiotic drive, a standard protocol was followed (Presgraves et al. 1997), and further details can be found in SI-A. Briefly, males are individually mated to ST-stock females. Males producing highly female biased broods are assumed to carry X^SR^. Their female offspring are therefore heterozygous for X^SR^ and when mated to ST-stock males, produce 50:50 X^SR^/Y: X^ST^/Y male offspring. These males are mated as before to ST-stock females and the female offspring from highly female biased broods are used to continue the stock. Over generations the SR phenotype has become more distinct as the stock maintenance procedure selects for female biased broods, and at the time of this experiment distortion typically produced ≥90% female offspring. The autosomes, Y-chromosome and mitochondrial genes are homogenised across the two stocks because the SR-stock maintenance involves backcrossing to ST-stock males and females. Males used in experiments are expected to be approximately 50:50 X^SR^/Y: X^ST^/Y as they inherit either an X^SR^ or X^ST^ chromosome from their mothers. For brevity, X^SR^/Y and X^ST^/Y males are hereafter referred to as SR and ST males, respectively.

The stock populations were kept at 25 °C, with a 12:12 h dark:light cycle and fed puréed sweetcorn twice weekly. Fifteen-minute artificial dawn and dusk periods were created by illumination from a single 60-W bulb at the start and end of the light phase. Experimental flies were collected from egg-lays placed in the stock population cages. Egg-lays consist of damp cotton-wool and excess puréed sweetcorn contained in a Petri dish. After eclosion, adult flies were measured for eyespan and thorax length using ImageJ (v1.46) and separated by sex prior to sexual maturity (<3 weeks after eclosion). Eyespan was defined as the distance between the outer tips of the eyes (Hingle et al. 2001). Thorax length was measured ventrally from the anterior tip of the prothorax along the midline to the joint between the metathoracic legs and the thorax (Rogers et al. 2008). At the time of testing, all flies were >6 weeks old and so had reached sexual maturity (Baker et al. 2003).

### Sperm allocation with variation in female quality

To test for differences in sperm allocation by SR and ST males, we examined sperm storage by females after a single mating. Males were tested with large and small females to test whether sperm allocation varied with female quality. ST and SR experimental, non-virgin males were taken from the SR-stock population (which produces both ST and SR males in a 50:50 ratio). They were held in 500 ml pots without access to females for at least 48 h prior to testing, so were not sperm or accessory gland product depleted, as testes and accessory glands are known to recover to full size within 24 h of mating (Rogers et al. 2005). Experimental males were placed at artificial dawn with either a large (eyespan ≥6 mm) or small (eyespan 4.1–5.2 mm) virgin female, isolated from the ST-stock population. Intermediate eyespan females were discarded. After a single mating, males were then kept in isolation for at least 48 h to allow the recovery of sperm number and accessory gland products, and subsequently mated to a female from the opposite size-class. Male genotypes were not known until after the experiment, so measures of sperm number (see below) were blind.

Mated females were anaesthetised on ice and their reproductive tracts were removed 4–6 h after mating. The spermathecae were isolated and placed on a glass microscope slide with 15 µl 4% formaldehyde and incubated on ice for 20 min. The spermathecae were then washed in a drop of phosphate buffered saline (PBS) and placed into 10 µl of dead stain (5% 2 mM propidium iodide diluted 1:20 in PBS, Sperm Viability kit, L-7011; Molecular Probes, Eugene, OR). A cover slip was placed over the sample and the spermathecae were gently crushed. Fluorescing dead sperm were counted at 1000× magnification under an oil immersion lens using a fluorescence filter. The total number of sperm was counted three times and an average taken (*r* > 0.94, *P* < 0.001). Most males (*N* = 62) were phenotyped through offspring sex-ratio counts, with a few genotyped using genetic markers (*N* = 5, see below).

In addition, a set of unmated females were dissected and their spermathecae were photographed at ×400 magnification using a monochrome microscope camera and QCapture Pro imaging software (v7.0). The area of the singlet and doublet spermathecae were measured using ImageJ (v2.0) by tracing the outline to give a longitudinal surface area, and an average was taken for the doublet.

### Sperm movement to the site of fertilisation

To examine sperm that migrated to the ventral receptacle (VR), the site of egg fertilisation, each male was mated once following a similar protocol as in the assays of sperm in the spermathecae. Dissections were performed 4–9 h after mating. The whole female internal reproductive tract was dissected into PBS and a cover slip placed over the sample. The total number of pouches that contained sperm as well as empty pouches in the VR were counted at 1000× magnification using an oil immersion lens. Three pouch counts per VR were made and an average taken (*r* > 0.86, *P* < 0.001). The presence of sperm was also recorded in the spermathecae. In a further experiment, females were mated as described and dissected two days after mating (51–58 h) and the VR examined as before. From the early period, 118 males were phenotyped through offspring sex-ratio counts, and 121 were genotyped using a genetic marker. From the later period, 191 males were phenotyped, and 54 were genotyped.

### Sperm allocation across sequential matings

To examine male sperm number over successive matings, a similar protocol was followed to that used to look at sperm in the spermathecae. In this experiment, we did not vary female size, but selected mid-sized females with eyespan between 5.25 and 5.95 mm (small and large eyespan females were excluded). At artificial dawn, males were introduced to a virgin female. After the male had mated to the first female, he was removed and placed with the second female, and subsequently with a third female. Mated females were dissected and the sperm stored in the spermathecae were counted, as in the previous assay (repeated count correlation *r* > 0.97, *P* < 0.001). Eight males were phenotyped and 38 males were genotyped. Lastly, a final experiment repeated this protocol but examined only the sperm transferred on a male’s third mating (repeated count correlation *r* > 0.95, *P* < 0.001). All 111 males were genotyped.

### Phenotyping and genotyping

After use in an experiment, males were classified as SR or ST using phenotypic offspring counts or genotypic INDEL markers. Phenotyped males were kept with three non-focal females for up to 4 weeks and egg-lays were collected twice weekly. Males were subsequently stored in ethanol at −20 °C. Males were classified as SR or ST through offspring counts by testing for deviations from a 1:1 sex ratio using *χ*^2^ tests on offspring counts greater than 10. Males with a brood sex-ratio diverging significantly (*P* < 0.05) from 1:1 and with ≥90% female bias were classified as SR. Males that did not deviate from a 1:1 brood sex-ratio were classified as ST. Males with a brood significantly different from 1:1 but with <90% female bias were further analysed using genotypic markers.

Alternatively, males were classified as SR and ST males using two INDEL markers, *comp162710* and *cnv395* (using methods described in SI-A). In both cases, allele sizes segregate into two categories—large and small—and a genotype can be assigned based on the size category. In laboratory samples from the SR-stock population, males with a small allele from either one of these markers are 83–90% likely to have an SR phenotype. An analysis of the error rates relating to the use of these genetic markers is provided in the SI-A.

### Statistical analysis

All tests were carried out in R version 3.31 (R Core Team 2016). To test if SR males differ in their rate of failure to deliver sperm to females, the presence or absence of sperm within the spermathecae (coded as 1 or 0) were fitted using general linear models (GLMs), or generalised linear mixed effects models (GLMMs), with a binomial error distribution. We also looked at the total number of sperm stored in the spermathecae in a GLM or GLMMs with a Poisson error distribution. Similarly, we tested if SR male sperm differs in the rate of transfer to the VR, both presence/absence (coded as 1 or 0) in GLMs with a binomial error distribution, and total number of sperm stored in a GLM with a Poisson error distribution.

Spermathecae and VR sperm count data were overdispersed, and so an observational level random effect (OLRE) was added (Harrison 2014), or a quasi-error distribution (binomial or Poisson) was used. These models included female size (large or small), mating order (1st, 2nd, or 3rd), male type (SR or ST) and their interactions as fixed effects. They also included male thorax length to control for male body size, as well as male eyespan to control for male quality, as male eyespan is strongly condition-dependent compared to other traits (David et al. 1998; Cotton et al. 2004a, b). Male thorax and eyespan had no consistent effect on sperm presence or number (see SI-B). GLMMs included male ID as a random effect to account for repeated measures.

Female spermathecae size, as well as the number of pouches in the VR, were analysed as functions of female size (large or small) in linear models. *P*-values were calculated with type II tests. Model tables and effect sizes are reported in SI-B.

## Results

### Sperm allocation with variation in female quality

Females were dissected 5.402 ± 0.527 (mean ± SD, *N* = 109) hours post-mating. 11% of females had no sperm in their spermathecae and SR had no effect on this failure rate (*χ*^2^_1_ = 0.039, *P* = 0.843, *N* = 109).

**Fig. 1:**
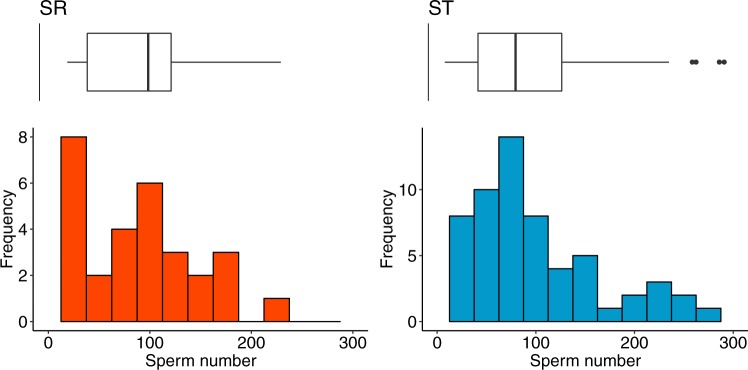
Number of sperm in female spermathecae after a single mating with either an SR (left) or ST (right) male. Upper: boxplots (first to third quartile) with median line and whiskers (1.5 IQR), and lower: frequency distribution of sperm counts

On average, females had 89.62 ± 69.17 (mean ± SD) sperm stored in their spermathecae (ignoring females with no sperm). There was no difference in the number of sperm stored in the spermathecae between SR (mean ± SD 87.76 ± 58.412, range 13–227) and ST males (90.38 ± 73.648, range 2–290, *χ*^2^_1_ = 0.267, *P* = 0.605, *N* = 97; Fig. 1). Large females had bigger spermathecae than small females, for both the singlet (*F*_1,52_ = 46.56, *P* < 0.001; mean ± s.e. large = 2.923 ± 0.051 mm^2^, *N* = 29, small = 2.410 ± 0.055, *N* = 25), and the doublet (*F*_1,62_ = 87.75, *P* < 0.001; mean ± s.e. large = 2.756 ± 0.035 mm^2^, *N* = 37, small = 2.224 ± 0.046, *N* = 27). But female size did not influence the number of sperm stored in the spermathecae (*χ*^2^_1_ = 0.47, *P* = 0.493, *N* = 97; Fig. SA1), nor did this depend on SR (male type x female size *χ*^2^_1_ = 0.071, *P* = 0.789, *N* = 97; Fig. SA1).

### Sperm movement to the site of fertilisation

In a second set of experiments, we measured sperm transferred to the ventral receptacle (VR), the site of egg fertilisation. Experimental females were dissected at two time points, either early shortly after mating (mean ± SD 5.672 ± 1.403 h, *N* = 239), or late two days after mating (mean ± SD 54.033 ± 1.53 h, *N* = 245). Sperm presence was recorded in the spermathecae as well as the VR. When sperm was present in the VR, it was also always present in the spermathecae (with a single exception). However, the reverse was not true. Of the females with sperm present in the spermathecae, the proportion of females with sperm also in the VR increased from the early (43.5%, 74/170) to late period (72.8%, 155/213; *χ*^2^_1_ = 35.1, *P* < 0.001). There was no effect of SR on the number of females with sperm in the VR in the early (*χ*^2^_1_ = 1.633, *P* = 0.201, *N* = 170) to the late period (*χ*^2^_1_ = 0.483, *P* = 0.487, *N* = 213).

**Fig. 2:**
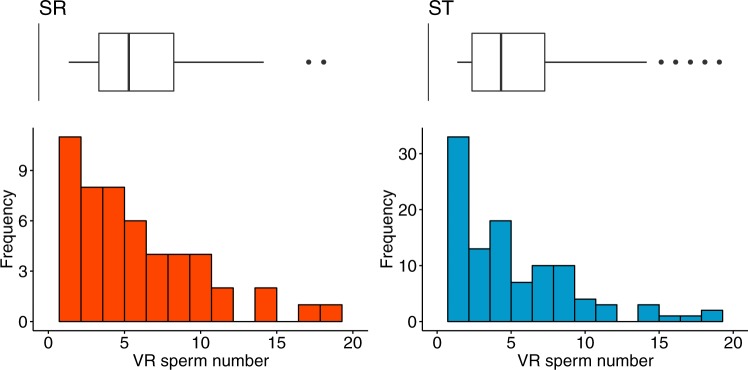
Proportion of pouches in the ventral receptacle (VR) filled with sperm after a single mating with an SR (left) or ST (right) male, two days after mating (late period). Upper: boxplots (first to third quartile) with median line and whiskers (1.5 IQR), and lower: frequency distribution of proportions

The number of sperm in the VR did not depend on SR in the early (F_1,68_ = 0.150, *P* = 0.285, mean ± SD SR = 4.0 ± 4.540, ST = 5.424 ± 6.018) or late period (*F*_1,149_ = 0.874, *P* = 0.351, mean ± SD SR = 5.940 ± 4.11, ST = 5.286 ± 4.094, Fig. 2). VR pouch number increased from small (mean ± s.e. 31.913 ± 0.374, *N* = 188) to large females (36.776 ± 0.328, *N* = 265, *F*_1,451_ = 94.224, *P* < 0.001). But there was no effect of female size on sperm number in the VR in the early (*F*_1,68_ = 0.790, *P* = 0.377) or late period (F_1,149_ = 0.025, *P* = 0.874), and no difference with SR in the early (male type x female size *F*_1,68_ = 0.0.653, *P* = 0.422) or late period (male type x female size *F*_1,149_ = 1.056, *P* = 0.306).

### Sperm allocation across sequential matings

In the penultimate experiment, males were mated sequentially to three different females (SR *N* = 11, ST *N* = 35). As in the previous experiments, SR had no effect on the failure rate to transfer sperm (*χ*^2^_1_ = 0.013, *P* = 0.908, *N* = 135). The failure to transfer sperm did not differ with the order of mating from first to third (*χ*^2^_1_ = 1.800, *P* = 0.406, *N* = 135), nor did this depend on SR (male type × mating order *χ*^2^_1_ = 0.721, *P* = 0. 697, *N* = 135).

**Fig. 3:**
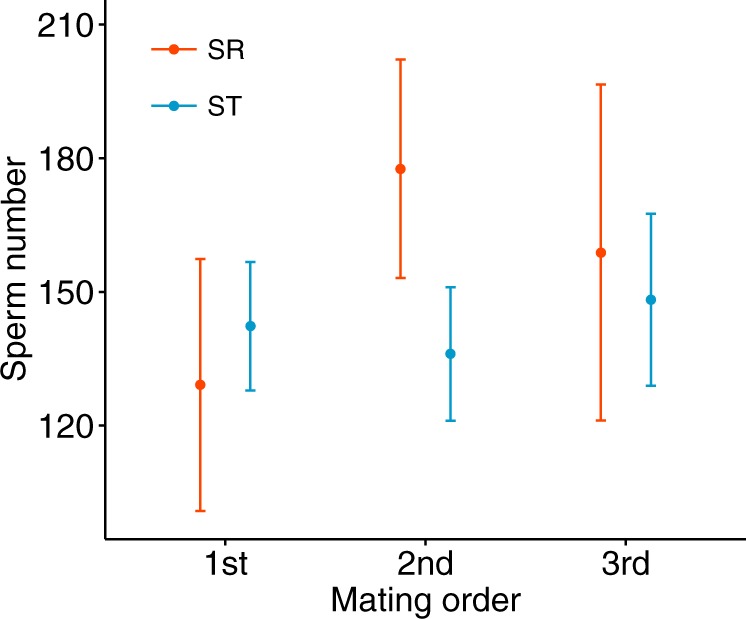
Number of sperm stored in the female spermathecae after an SR (left) or ST (right) male’s first (SR *N* = 9, ST *N* = 28), second (SR *N* = 8, ST *N* = 27) or third mating (SR *N* = 6, ST *N* = 25), showing mean ± s.e

There was also no effect of SR on the number of sperm stored in the spermathecae (*χ*^2^_1_ = 1.372, *P* = 0.241, *N* = 103, Fig. 3). Sperm number did not change with the order of mating from first to third across contiguous successful matings (*χ*^2^_1_ = 0.198, *P* = 0.906, *N* = 103), nor was there an interaction of SR with mating order (*χ*^2^_1_ = 2.415, *P* = 0.299, *N* = 103). The pattern of results remained the same when looking across all successful matings (i.e., including all males with at least one mating where sperm were present, all *P* > 0.05, *N* = 118). In a final experiment, a larger sample of males (SR *N* = 19, ST *N* = 64) were mated three times with sperm counts taken at the final mating. Once again, sperm number in the spermathecae did not depend on SR (*χ*^2^_1_ = 0.046, *P* = 0.830, mean ± SD SR 28.158 ± 23.217, ST 28.746 ± 25.548).

## Discussion

Male meiotic drive typically involves the dysfunction of gametes. In the extreme, half the gametes are disabled (Burt and Trivers 2006; Price and Wedell 2008). Whilst this is beneficial to the drive element itself because it excludes non-carrier sperm, in many systems it leads to a reduction in the fertility of drive males (Peacock and Erickson 1965; Jaenike 1996; Atlan et al. 2004; Wilkinson et al. 2006; Angelard et al. 2008; Price et al. 2012; Pinzone and Dyer 2013), particularly under conditions of sperm competition (Wilkinson and Fry 2001; Atlan et al. 2004; Angelard et al. 2008; Price et al. 2008). In some cases, drive has been demonstrated to result in a reduction in the amount of sperm transferred to females at mating (*D. melanogaster*: Peacock and Erickson 1965; *D. simulans*: Angelard et al. 2008; *D. pseudoobscura*: Price et al. 2008). Contrary to these observations, we find no evidence that SR males transfer reduced sperm numbers in *T. dalmanni*. In female spermathecae, the long-term primary storage organs, sperm numbers were not different between those of females mated to SR and ST males. Likewise, transfer of sperm to the ventral receptacle (VR), a small organ to which sperm migrate prior to use in fertilisation, was also similar for SR and ST male sperm, over both short and longer time frames (after two days). Furthermore, sperm numbers in storage were similar for females mated to SR and ST males even after males had mated multiple times. Across all sampled organs and time points, SR males delivered comparable amounts of sperm to ST males per ejaculate.

Sperm are initially delivered to females in a spermatophore. We did not measure sperm numbers directly from the spermatophore, as it is very small and the sperm within it are compact and cannot be distinguished individually (Kotrba 1996; Rogers et al. 2006; Harley et al. 2013). This raises the possibility that SR and ST sperm numbers differed in the spermatophore, but there is a capacity limit on transfer to storage in the spermathecae, thereby imposing equality even though SR males deliver half-size ejaculates. We think this is highly unlikely. Firstly, the range of sperm numbers recorded in the spermathecae is immense, with values exceeding 300 and no difference between SR and ST male means (88 and 90 respectively) and frequency distributions (Fig. 1). Second, while spermathecae vary in size, and scale with female size, there is no evidence that large females—with large storage capacity—store more sperm than smaller females (Fig. SA1). The hypothesis of a capacity limit does not appear to explain the similarity in SR and ST sperm counts. Our measure of sperm in the spermathecae might also be explained by adaptive compensation in ejaculate composition, rather than sperm number. The evolution of seminal fluid proteins could enable SR males to get proportionally more sperm into storage, despite transferring fewer sperm overall. However, this is a less persuasive explanation for why we find no difference between SR and ST male sperm numbers in storage, as there is no clear reason why ST males would not also be able to provide a similar cocktail of accessory gland products.

These findings in *T. dalmanni* challenge the conventional assumption that drive acts like a genetic disease that causes disruption of normal male reproductive activity. This static view ignores the possibility of adaptive responses to drive that ameliorate its negative effects and restore organismal fitness. There is considerable theoretical work on optimal ejaculate expenditure that is relevant to understanding investment strategies in drive males (Parker 1998; Tazzyman et al. 2009; Engqvist 2012). Given that sperm competition follows a fair raffle and males lack information about previous or future female mating patterns, then all males face the same risk and intensity of sperm competition (Parker 1998). Under these conditions, a male’s optimal ejaculate size per mating is predicted to be independent of the resources that he has available to allocate to reproduction (Tazzyman et al. 2009). If we consider drive males to have half the resources for reproduction compared to wildtype males, then they are expected to produce ejaculates of similar size (in order to be able to optimise their success in sperm competition) but simply mate less often (Tazzyman et al. 2009). A subsequent modelling approach considered competition between two types of males, fertile and sub-fertile (i.e., wildtype and drive), and reached broadly similar conclusions but more explicitly considered how sperm allocation varies with the degree of multiple mating of sub-fertile males (Engqvist 2012). Under high rates of female re-mating, as is typical in *T. dalmanni*, there are limited advantages to drive males of dividing their reproductive resource amongst females, as they rarely encounter females who have mated infrequently and constitute arenas of low sperm competition. The model predicts drive males should invest a similar amount or even more per ejaculate, though the predicted difference is expected to be small in many cases and unlikely to be detected easily (Engqvist 2012). Our results suggest that ejaculate size does not differ between SR and ST males, even after multiple mating. However, the experiments do not test SR males under conditions that they are likely to encounter in the wild. In particular, males that hold harems mate with multiple females during a short period at dawn before they disperse (Wilkinson et al. 1998; Chapman et al. 2005; Cotton et al. 2015). Further investigation is needed of how SR males perform when they have a limited time in which to engage with females, and to see whether they can maintain their sperm delivery in the longer term when there are repeated mating opportunities across days.

Such a response may occur if alleles that enable increased investment in sperm production become physically linked with the drive element. Drive elements are often associated with regions of low recombination such as inversions (James and Jaenike 1990; Johns et al. 2005; Dyer et al. 2007, 2013; Reinhardt et al. 2014). This allows drive and insensitive responder to remain together, as well as facilitating the accumulation of modifiers of drive that enhance transmission (Hartl, 1975; Larracuente and Presgraves, 2012). The structure of the driving X chromosome is not known in *T. dalmanni*, except that one or more inversions cover a large fraction of the chromosome, resulting in low recombination and reduced gene flow between X^SR^ and X^ST^ (Johns et al. 2005; Paczolt et al. 2017). Genetic linkage between the drive element and alleles beneficial to its transmission constitutes a plausible mechanism by which SR males could increase investment in sperm production.

Models of X-linked meiotic drive evolution predict that a stable equilibrium frequency of drive is reached under a wide range of fertility and viability costs imposed on male and female carriers (Edwards 1961; Hamilton 1967; Curtsinger and Feldman 1980). In particular, when female drive homozygotes suffer markedly, there is negative frequency-dependent selection against drive that can balance its transmission advantage (Curtsinger and Feldman 1980). Further complications follow from distortion in the population sex ratio reducing the frequency of multiple mating and hence the degree of sperm competition, which in general favours a higher frequency of drive at a balanced polymorphism (Taylor and Jaenike 2002). More recent theory has considered adaptive change in the host organism in response to the spread of drive. Research has centred on the idea that polyandry (i.e., the degree of multiple mating) acts as a female adaptation to reduce the fertilisation success of sperm from drive males, on the assumption that they are weak sperm competitors (Haig and Bergstrom 1995; Zeh and Zeh 1996). Polyandry should reduce the likelihood that offspring inherit the drive allele and thereby raise their fitness. Modelling shows that the co-evolution of female polyandry and meiotic drive causes a reduction in the population frequency of drive (Holman et al. 2015). In contrast, the maintenance of sperm allocation per ejaculate in drive males acts in the opposite direction. It would increase male fertility, not only to the benefit of the whole genome, but also to the benefit of the SR selfish genetic element. Though there are likely to be trade-offs associated with the compensatory delivery of greater sperm numbers per ejaculate (e.g., affecting the mating rate or some aspect of viability), we predict that the net effect would be an increase in the equilibrium frequency of drive. In addition, there may be less advantage to female polyandry if the number of sperm delivered offsets any sperm competition disadvantage of drive male ejaculates. There is some evidence that SR males do nonetheless perform poorly in competition with ST male ejaculates (Wilkinson et al. 2006), however the extent to which polyandry impacts on the transmission frequency of drive remains to be established. These different forms of selection, operating on individual males and females as well as at the level of the selfish genetic element, need to be considered in a full theoretical analysis, to understand how they alter the spread and equilibrium frequency of drive under different demographic and ecological conditions.

Adaptive change to ejaculate allocation seems unlikely to be found in systems in which drive has arisen recently (e.g., SR in *D. simulans*, Derome et al. 2004) or persists at such a low frequency that it exerts little selective effect on the host organism (e.g., SR in *D. recens* and *D. quinaria*, Jaenike 1996). This is not the case for X^SR^ in *T. dalmanni* where the current form of drive is estimated to be around 0.75 million years old (Paczolt et al. 2017). This may under-estimate the history of drive in the *Teleopsis* group as it is present in the sister species *T. whitei*, that diverged around 3.5 million years ago (Christianson et al. 2005; Swallow et al. 2005). In addition, X^SR^ is not rare, with a frequency of ~20% across many populations of *T. dalmanni* (Wilkinson et al. 2003; Cotton et al. 2014; Paczolt et al. 2017). The long-term persistence of drive at a significant frequency in this lineage seems likely to have created a selective environment favouring adaptive changes to tolerate its presence. Other older systems, such as SR in *D. pseudoobscura* which is estimated to be 1 million years old (Kovacevic and Schaeffer 2000), and the *t* haplotype in house mice which has persisted for over 1.5 million years (Hammer and Silver 1993), do not present evidence for a similar adaptive response in sperm allocation. In *D. pseudoobscura*, drive males transfer fewer than half the number of sperm in a single mating compared to wildtype males (Beckenbach 1978; Price et al. 2008) and their sperm are additionally weak in competition with wildtype sperm (Price et al. 2008, 2014). In house mice, the number of sperm transferred to the female uteri is unaffected by drive; even sterile homozygous drive males transfer large quantities of sperm (Tessler and Olds-Clarke 1981). However, drive male sperm motility is reduced sufficiently to impact on transport through the oviduct and to interfere with fusion with the oocyte (Olds-Clarke 1997). Unsurprisingly, drive males are poor sperm competitors as measured by the number of offspring sired (Manser et al. 2011; Sutter and Lindholm 2015). Furthermore, males that carry the *t* haplotype do not appear to adopt any other alternative reproductive tactics in behavioural or morphological traits, including testis size (Sutter and Lindholm 2016). It is not clear why these deleterious effects of drive have not led to the evolution of compensatory mechanisms. Both of these drive systems are associated with inversions. In *D. pseudoobscura* three non-overlapping inversions cover around half of the X chromosome (Sturtevant and Dobzhansky 1936; Kovacevic and Schaeffer 2000) whereas the *t* haplotype comprises four major inversions that completely suppresses recombination across the entire length of chromosome 17 (Artzt et al. 1982; Hammer et al. 1989). These linked regions present a large mutational target in which linked genes that alter ejaculate allocation in drive males could occur. For instance, many recessive lethal mutations have accumulated in this region, forming distinct haplotypes (Silver 1985). It is possible that the low frequency and patchy distribution of the *t* haplotype in wild populations limits selection on linked genes that enable changes to sperm allocation (Ardlie and Silver 1998). However, in *D. pseudoobscura*, SR exists along a latitudinal cline, reaching high and stable frequencies of up to 30% in its southern reach (Price et al. 2014). It is interesting to note that neither system of drive is associated with suppressors, as there is no genetic resistance in *D. pseudoobscura* (Policansky and Dempsey 1978), and suppressors of the *t* haplotype do not appear to be widespread (Ardlie and Silver 1996). These patterns suggest that there may only be weak selection for genes controlling compensatory mechanisms, either in sperm allocation or suppressors of drive.

It is generally assumed that, relative to wildtype males, drive males produce fewer viable sperm, have reduced fertility and are worse sperm competitors. However, this neglects the possibility that drive males have adapted to their sub-fertile condition. Theoretical models that examine the evolution of male ejaculate allocation do not predict that drive males invest less per ejaculate than do wildtype males. We find evidence for such an adaptive compensation, as drive male ejaculates are equivalent to those of wildtype males in terms of the number of sperm delivered to female sperm storage organs. This compensation also applies under multiple mating, as SR males were equivalent to ST males even after a third mating. We also show that SR male sperm are as capable as ST sperm of migrating from long-term storage (spermathecae) to the site of fertilisation (the ventral receptacle). Further work is needed to ascertain exactly how drive males are able to compensate for failed spermatogenesis, and whether this trades-off against investment in other important aspects of male fertility such as the costly non-sperm components of male ejaculate and the production of primary and secondary sexual traits.

### Data archiving

Raw data from the manuscript is archived in the Dryad Digital Repository, 10.5061/dryad.cf0468k.

## Electronic supplementary material

Supplementary Information A: Extended methods and figures

Supplementary Information B: Model tables and effect sizes
