# Supplementary material for: Ejaculate sperm number compensation in stalk-eyed flies carrying a selfish meiotic drive element
Source: Heredity (Edinb). 2018 Nov 22;122(6):916–26. doi: 10.1038/s41437-018-0166-y (PMC6781104; doi:10.1038/s41437-018-0166-y)
Supplement: Supplementary file 1 — Supplementary Information A: Extended methods and figures [file 41437_2018_166_MOESM1_ESM.pdf]

Compensation of ejaculate sperm number in male stalk-eyed flies carrying a selfish meiotic drive element

Meade, L.C., Dinneen, D., Kad, R., Lynch, D.M., Fowler, K. & Pomiankowski, A.

Supplementary Information A: Extended methods and figures

Contents

SI-A1 Sperm storage in small and large females 3

SI-A2 Stock source and maintenance 4

SI-A2.1 Standard stock population 4

SI-A2.2 Sex-ratio meiotic drive stock population 4

SI-A3 The use of microsatellite and INDEL markers to detect sex ratio meiotic drive in *Teleopsis dalmanni* 6

SI-A3.1 Introduction 6

SI-A3.2 Methods 7

SI-A3.3 Results 9

SI-A3.4 Conclusion 12

SI-A1 Sperm storage in small and large females

In the main text we report on sperm storage in the female spermathecae after mating to an SR or an ST male. Females were either small (eyespan 4.1 – 5.2 mm) or large (eyespan $\geq$6 mm). Spermathecae size scales with female size, but female size did not influence the number of sperm stored in the spermathecae (Fig. SA1).

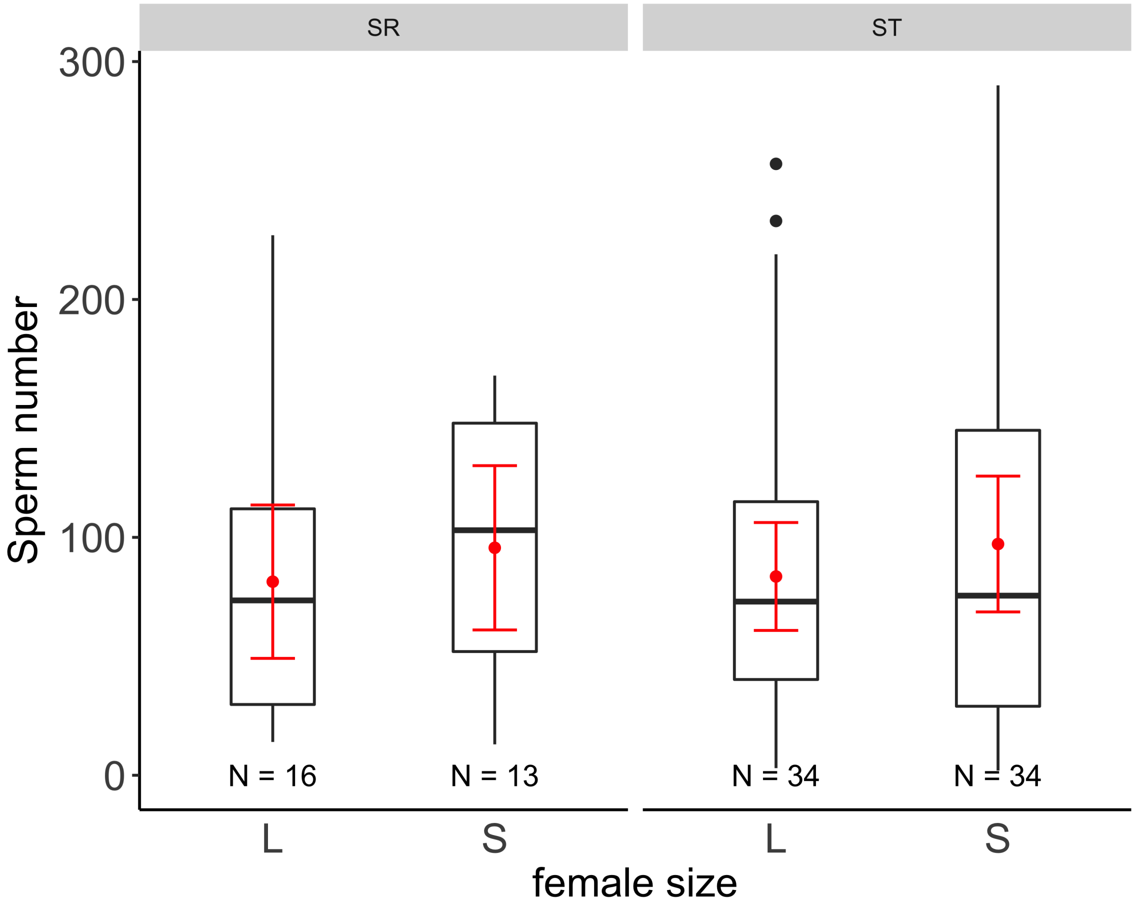

Figure SA1: Number of sperm stored in large (L; eyespan $\geq$6 mm) or small female's spermathecae after mating with an SR (pair of plots on the left) or ST (pair of plots on the right) male. Boxplots (first to third quartile) with median line and whiskers (1.5 IQR), and mean ± s.e. (red points and lines). Female size did not influence number of sperm stored (P = 0.493), and this did not depend on SR (male type x female size P = 0.789).

SI-A2 Stock source and maintenance

SI-A2.1 Standard stock population

The standard wildtype stock (ST-stock) population was created from collections in 2005, as described in the main manuscript. This population has been regularly monitored and does not contain meiotic drive and carries non-distorting standard X chromosomes (X^{ST}).

SI-A2.2 Sex-ratio meiotic drive stock population

Flies were collected in 2012 (by A. Cotton and S. Cotton) from the Ulu Gombak valley to create a sex ratio meiotic drive stock (SR-stock) population. To establish and maintain a stock with meiotic drive, a standard protocol was followed (Presgraves et al. 1997). Briefly: wild males (of unknown genotype) were mated to ST-stock females and their offspring (F1) were collected. When an F1 brood was female biased (80% female, > 10 offspring), it was assumed that the father was a carrier of the sex-ratio distorting X^{SR} chromosome, so that the F1 female offspring had genotype X^{SR}/X^{ST}.

When sexually mature (> 4 weeks, Baker et al. 2003), F1 X^{SR}/X^{ST} females were mated with ST-stock males and their offspring (F2) were collected. X^{SR}/X^{ST} females and ST-stock males were housed in cage populations of $\sim$100 individuals at 1:1 sex-ratio. Male F2 offspring are expected to be 50:50 X^{SR}/Y:X^{ST}/Y as they inherit either an X^{SR} or X^{ST} chromosome from their mother. F2 males were subsequently mated to ST-stock females to identify those males carrying X^{SR}, and the process repeated.

Even though there was error in the assignment of individuals as carriers of X^{SR}, the process maintains the X^{SR} chromosome in this stock. Over generations the SR phenotype has become more distinct as the stock maintenance procedure selected for female biased broods, so most SR-stock males now produce only female offspring, or at least > 95% female biased broods. Note that because the SR-stock maintenance involves back-crossing to ST-stock males and females, the autosomes, Y-chromosome and mitochondrial backgrounds are homogenised across the two stocks. For brevity, we hereafter refer to X^{SR}/Y and X^{ST}/Y males as X^{SR} and X^{ST} males respectively.

The stock populations were kept at 25°C, with a 12:12 h dark:light cycle and fed puréed

sweetcorn twice weekly. Fifteen-minute artificial dawn and dusk periods were created by illumination from a single 60-W bulb at the start and end of the light phase.

SI-A3 The use of microsatellite and INDEL markers to detect sex ratio meiotic drive in *Teleopsis dalmanni*

SI-A3.1 Introduction

To conduct efficient experiments on drive in the stalk-eyed fly *Teleopsis dalmanni*, it is vital to have a convenient and accurate method of distinguishing drive from wildtype males. A predominant conventional method is through offspring counts and performing χ^2 tests of significance on deviations from a 1:1 sex ratio. However, this labour-intensive and time consuming method is limited in multiple ways. Firstly, a sample of at least 10 offspring is needed perform a χ^2 test (Cochran 1952), so it is not possible to assign a phenotype to less prolific males. Furthermore, various factors may influence brood sex-ratio independent of drive, causing false identification of drive and wildtype males. For example, selection may operate on larval survival and thus alter sex ratios. This may lead to variation in wildtype male brood sex-ratio, sufficient to emulate drive. Brood sex-ratios are also of limited use in identifying females that are heterozygous or homozygous for meiotic drive. Finally, and importantly, there are many situations in which there is no opportunity for males to sire offspring, for example, when collecting flies in the field, or when there is a need to use laboratory males in experiments as soon as they are sexually mature or as virgins. These factors severely limit the utility of offspring counts as a means of identifying individuals that carry meiotic drive.

An alternative approach is to use genetic markers that can reliably predict the phenotype of individuals. The task of finding useful markers is feasible because the sex ratio distortion X chromosome (X^{SR}) shows widespread divergence from the standard X chromosome (X^{ST}) (Christianson et al. 2011; Cotton et al. 2014; Reinhardt et al. 2014; Paczolt et al. 2017). Furthermore, recombination between X^{SR} and X^{ST} is rare or absent (Johns et al. 2005; Paczolt et al. 2017). A number of X-linked microsatellite markers were identified for *T. dalmanni* which showed association with meiotic drive (Johns et al. 2005). A further investigation using wild collected flies found that only one of the four microsatellites, *ms395*, was predictive of the drive phenotype in *T. dalmanni*, albeit with a rather high error (Cotton et al. 2014). Here we further investigate locus *ms395*, along with three additional X-linked INDEL markers, *comp162710*, *cnv395* and *cnv125*. Using data from laboratory reared flies, we assess these markers for their

predictive power of male phenotype (ST or SR). When samples carrying particular allele all tend to exhibit a single phenotype, this demonstrates high predictive power. The consistency of each marker can be additionally informative about its usefulness in detecting X^{SR}. A marker is consistent when phenotypes are represented by a single allele size.

SI-A3.2 Methods

SI-A3.2.1 Phenotype assignment

To produce offspring to determine male brood sex-ratio, males were kept with three non-focal females for up to 4 weeks and egg-lays, consisting of damp cotton-wool and excess puréed sweetcorn contained in a Petri dish, were collected twice weekly. Eggs were allowed to develop into pupae and offspring were collected and sexed until no more offspring emerged from the egglay. Males were subsequently stored in 100% ethanol at -20°C.

Significant ($P < 0.05$) deviation from a 1:1 sex ratio was tested for using χ^2 tests on offspring counts with a minimum of 10 offspring. Males that had a significantly female biased brood sex-ratio of $\geq$ 80% were categorised as SR. Males were otherwise classed as ST.

SI-A3.2.2 Genotyping

Three X-linked INDEL markers (*comp162710*, *cnv395* and *cnv125*) were developed from sequenced drive and non-drive populations from Kanching (3° 18'N 101° 37'E) and Ulu Gombak valley, Peninsular Malaysia (J. Reinhardt, K. Paczolt and G.S. Wilkinson, *personal communication*). The product sizes of these markers clearly segregate into two categories—small and large. These alleles were reported to co-segregate with ST and SR phenotype males: for *comp162710* ST segregates with the large allele (286 bp) and SR with the small allele (201 bp), for *cnv395* ST segregates with the large allele (362 bp) and SR with the small allele (330 bp), and for *cnv125* ST segregates with the large allele (358 bp) and SR with the small allele (129 bp). The *ms395* locus has previously been shown to have an association with the drive phenotype in wild males (Cotton et al. 2014), where large *ms395* alleles (>218 bp) are associated with female-biased broods. Primer sequences can be found in Table SA1.

A standard protocol was followed to extract DNA (Bruford et al. 1998). For each sample,

Table SA1: Primer sequences for microsatellite *ms395* and INDELs *comp162710*, *cnv395* and *cnv125*

locus	forward	reverse
ms395	[HEX]CGAGTAGAGCACTTTGAAGATACA	TTGCGGTTGTAGAAGTTTGC
comp162710	[6-FAM]CGTGTCCGCATTTATACCAC	GGTAGGCTTGTTCTAACGGC
cnv395	[HEX]TGAGAGAAAGGGCAGCAAAG	GTGAGTGCAGCCAATAGTGT
cnv125	[6-FAM]AGGAACGCAATGCCTAGTTG	TGGACTTGGGTTTACTTGGG

half a thorax was crushed and digested in 250μl digestion solution (20mM EDTA, 120mM NaCl, 50mM Tris-HCL, 1% SDS, pH 8.0) and 10μl proteinase K (10mg ml^{-1}), and the samples incubated for $\sim$12hrs at 55°C. Proteins were precipitated out with 300μl of 4M ammonium acetate and spun at 13000rpm for 10min. The supernatant was aspirated into 1ml absolute ethanol to precipitate out the DNA, which was pelleted by spinning at 13000rpm for 10min. The DNA pellet was washed in 500ml of 70% ethanol and allowed to dry before being stored in 50μl T10 E0.1 buffer at -20°C. Primers were arranged into two multiplexes (*ms395* and *comp162710*, *cnv395* and *cnv125*) and PCR reactions were performed on a 2720 Thermal Cycler (Applied Biosystems, Woolston, UK) in 2μl samples, containing 1μl QIAGEN Mastermix (QIAGEN, Manchester, UK), 1μl Primer mix and 1μl DNA (dried). All primers were at a 0.2μM concentration. PCR reactions had an initial denaturing stage of 95°C for 15min followed by 45 cycles of 94°C for 30sec, 60°C for 1min 30sec and 72°C for 1min 30sec. This was completed by an elongation step of 60°C for 30min. The Applied Biosystems ABI3730 Genetic Analyzer was used to visualise the microsatellites, with a ROX500 size standard. GENEMAPPER 4.0 was used to assign microsatellite allele sizes. Sequencing work was carried out at the NERC Biomolecular Analysis Facility at the University of Sheffield.

SI-A3.2.3 Statistical analysis

Analyses were carried out in R version 3.31 (R Core Team 2016). Only males that produced at least 10 offspring were included in the analyses. The relationship between allele size and brood sex-ratio for each X-linked locus was examined using generalised linear models (GLMs). Offspring counts were analysed as proportion data (total female off-spring, total male offspring) in binomial GLMs. These models assess sex ratio bias, while accounting for brood size. The

data was over-dispersed, so models were fitted with a quasi-binomial error distribution and a logit link function. *ms395* allele size was included as a nominal variable, split into groups of 10 base pairs, as in Cotton et al. (2014). The allele sizes of the three INDEL markers segregate into two distinct size groups (Table SA2), and so allele size for *comp162710*, *cnv395* and *cnv125* were split into two groups of small and large alleles. We subsequently split *ms395* alleles into large and small depending in whether they were > 218 or not (Cotton et al. 2014), and for each locus we examined the frequency distribution of allele size groups between brood sex ratio phenotype categories using Fisher's exact test.

Lastly, allele size groups were evaluated for their consistency at predicting phenotype. Loci are consistent within a phenotype when most individuals of that phenotype carry the same size allele. Consistency was calculated as the absolute value of the frequency of small alleles (p) minus the frequency of large alleles (q), $|p - q|$, for each phenotype. A value of 0 indicates complete inconsistency, where a phenotype is equally likely to carry a small or large allele. A value of 1 indicates complete consistency, where all members of a phenotype category carry the same size allele.

SI-A3.3 Results

From laboratory stocks, 35.8% of males produced significantly sex ratio biased broods (229/639). Families with significant sex-ratio distortion were mostly female biased (222), but a smaller number were significantly male biased (7). We have no reason to believe that a male biased sex-ratio is actually genetically distinct from an ST phenotype. 66 males with female biased broods, 7 males with male biased and 132 males with unbiased broods had allele size information for at least one marker (*ms395*, *comp162710*, *cnv395*, *cnv125*). After applying the criteria for defining male phenotype category, 174 males were classed as ST and 31 as SR (Fig. SA2).

SI-A3.3.1 Brood sex-ratio and allele size

The relationship reported previously by Cotton et al. (2014) with *ms395* allele size and brood sex-ratio in wild males was also also found here in laboratory samples ($F_{3,178}$ = 48.076, P < 0.001, Fig. SA2a). Similarly, *comp162710* ($F_{1,195}$ = 401.35, P < 0.001, Fig. SA2b) and *cnv395* ($F_{1,186}$ = 150.18, P < 0.001, Fig. SA2c) allele size also exhibited a relationship with brood

Table SA2: Allele sizes for each locus (microsattelite *ms395* and INDELs *comp162710*, *cnv395* and *cnv125*) segregate into two size groups—small and large.

locus	small (bp)	large (bp)
ms395	197 – 206	227 – 247
comp162710	201	286 – 287
cnv395	331	360 – 370
cnv125	128	359 – 361

sex-ratio. *cnv125* showed no relationship with brood sex-ratio ($F_{1,114}$ = 1.614, P = 0.207, Fig. SA2d). For each locus, alleles segregated into two groups of small and large alleles (Table SA2). Small and large alleles were not distributed randomly between phenotypes (Table SA3) for *ms395* (Fisher's exact test P < 0.001), *comp162710* (P < 0.001), *cnv395* (P < 0.001) or *cnv125* (P = 0.012). Each allele size (small/large) was highly predictive of either an ST or an SR phenotype (Table SA3) for *ms395* (ST: 96%; SR: 92%), *comp162710* (ST: 98%, SR: 90%) and *cnv395* (SR: 98%, SR: 83%). For *cnv125*, a large allele was highly predictive of an ST phenotype (97%), however, a small allele was a poor predictor of both ST (78%) and SR (22%).

SI-A3.3.2 Allele size consistency

Allele size consistency ranges from 0 (phenotype represented by either allele) to 1 (phenotype represented by single allele). Allele sizes were highly consistent for ST males for all markers except *cnv125* (Table SA3, Fig. SA2). Almost all ST males carried single allele size (small/large) for *ms395* (consistency = 0.99, N = 161), *comp162710* (consistency = 0.96, N = 167) and *cnv395* (consistency = 0.95, N = 164), while for *cnv125*, ST males could carry either allele (consistency = 0.29, N = 96). Overall, allele sizes within SR were also consistent, and remained biased towards the alternative allele size group to ST samples (Table SA3, Fig. SA2), for *ms395* (consistency = 0.33, N = 18), *comp162710* (consistency = 0.79, N = 29) and *cnv395* (consistency = 0.74, N = 23), as well as for *cnv125* (consistency = 0.89, N = 19).v

Amplification success varied across the four loci. For the 211 males examined, 85% for *ms395*, 93% for *comp162710*, 89% for *cnv395* and 55% for *cnv125*. Where samples failed to amplify for *comp162710* (N = 15), all samples also failed for *ms395*, indicating minor technical issues because these loci were amplified in a multiplex. In contrast, all 15 amplified for *cnv395*.

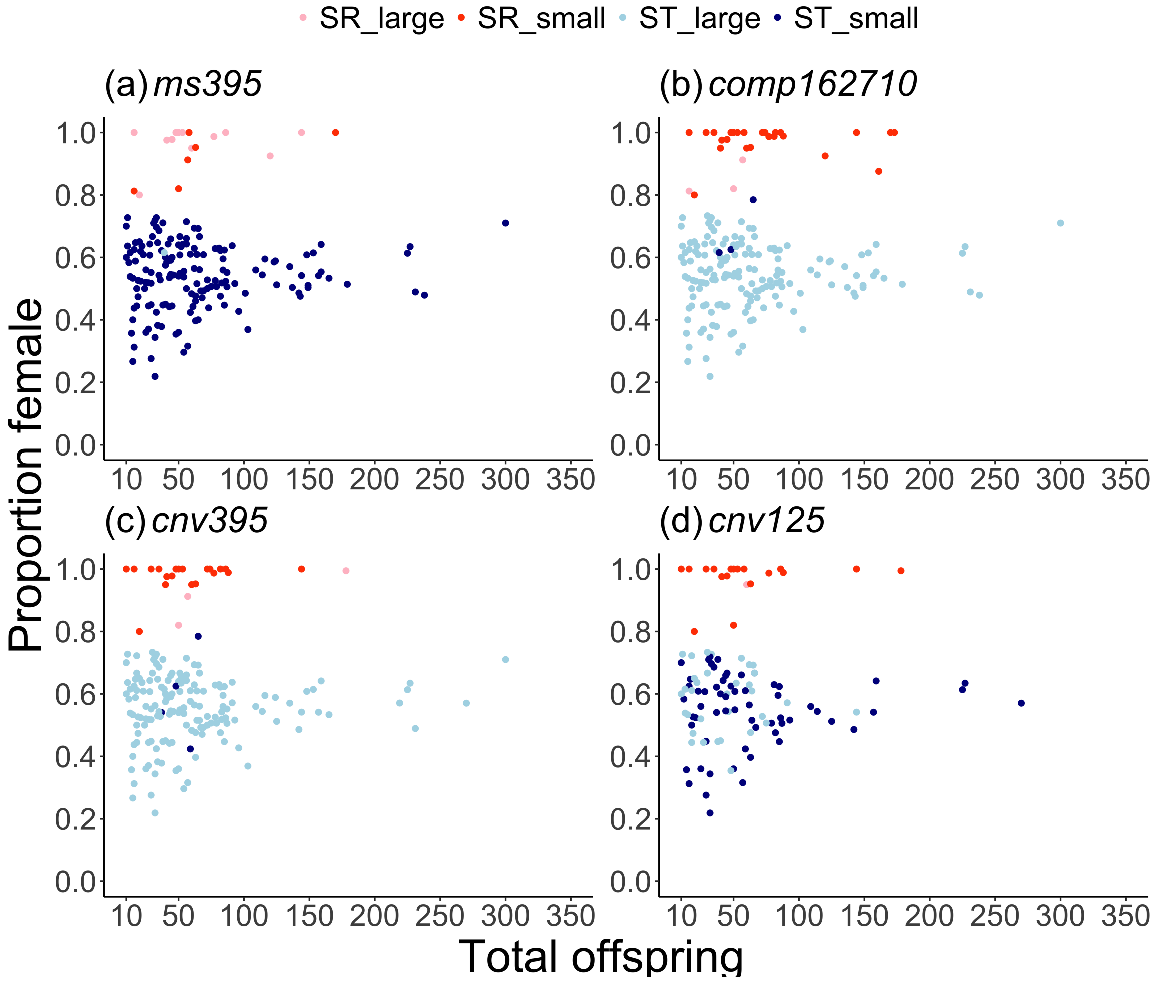

Figure SA2: Plot of brood sex-ratios, given as proportion of females, against total offspring. Males are categorised SR (red and pink; P < 0.05, and brood sex-ratio > 0.8) or as ST (dark and light blue), using χ^2 tests on offspring counts greater than 10 testing for significant (P < 0.05) deviations from a 1:1 sex ratio. Males carry either a large (light colour) or small (dark colour) allele for each marker (a, *ms395* N = 179; b, *comp162710* N = 196; c, *cnv395* N = 186; d, *cnv125* N = 115).

Table SA3: Allele distribution between phenotypes in laboratory samples. Allele sizes for each locus (microsattelite *ms395* and INDELs *comp162710*, *cnv395* and *cnv125*) segregate into two size groups—small and large. Allele size consistency indicates whether each phenotype category (ST or SR) tends to be represented by one or both allele size groups. A consistency value of 0 (not consistent) indicates that samples may carry an allele of either size, while a value of 1 (consistent) means samples are represented by a single allele size group.

locus	allele	ST	SR	%ST	%SR
	small	160	6	96	4
ms395	large	1	12	8	92
	allele size consistency	*0.99*	*0.33*		
	small	3	26	10	90
comp162710	large	164	3	98	2
	allele size consistency	*0.96*	*0.79*		
	small	4	20	17	83
cnv395	large	160	3	98	2
	allele size consistency	*0.95*	*0.74*		
	small	62	18	78	22
cnv125	large	34	1	97	3
	allele size consistency	*0.29*	*0.89*		

SI-A3.4 Conclusion

To be of value for identifying individuals which carry X^{SR}, markers must associate with brood sex-ratio and be a reliable predictor of a phenotype category. Here we evaluated four X-linked markers (one microsatellite and three INDEL markers) and found that three of four markers associate with brood sex-ratio, and all have some predictive value (Table SA3). All loci are good at predicting ST (96 – 98%), while *comp162710*, *ms395* and *cnv395* also reliably predict an SR phenotype (83 – 92%). *cnv125* is uninformative because whilst almost all SR males have the same sized allele, most ST males also have this allele. We can conclude that *cnv125* is not useful for assigning SR and is not a worthwhile marker to be used for laboratory analyses.

Furthermore, markers *ms395*, *comp162710* and *cnv395* amplified well, while amplification rates for *cnv125* were poor in comparison.

References

Baker, R. H., Denniff, M., Futerman, P., Fowler, K., Pomiankowski, A., and Chapman, T. (2003). Accessory gland size influences time to sexual maturity and mating frequency in the stalk-eyed fly, *Cyrtodiopsis dalmanni*. *Behavioral Ecology* **14**:607–611. doi: 10.1093/beheco/arg053.

Bruford, M. W., Hanotte, O., Brookfield, J. F. Y., and Burke, T., (1998). Multi and single locus DNA fingerprinting. Pages 287–336 *in* A. R. Hoelzel, ed. Molecular Analysis of Populations: A Practical Approach. IRL Press, Oxford, 2nd edition.

Christianson, S. J., Brand, C. L., and Wilkinson, G. S. (2011). Reduced polymorphism associated with X chromosome meiotic drive in the stalk-eyed fly *Teleopsis dalmanni*. *PLoS ONE* **6**:e27254. doi: 10.1371/journal.pone.0027254.

Cochran, W. G. (1952). The $\chi 2$ test of goodness of fit. *Annals of Mathematical Statistics* **23**:315–345. doi: 10.2307/2236678.

Cotton, A. J., Földvári, M., Cotton, S., and Pomiankowski, A. (2014). Male eyespan size is associated with meiotic drive in wild stalk-eyed flies (*Teleopsis dalmanni*). *Heredity* **112**:363–9. doi: 10.1038/hdy.2013.131.

Johns, P. M., Wolfenbarger, L. L., and Wilkinson, G. S. (2005). Genetic linkage between a sexually selected trait and X chromosome meiotic drive. *Proceedings of the Royal Society B: Biological Sciences* **272**:2097–2103. doi: 10.1098/rspb.2005.3183.

Paczolt, K. A., Reinhardt, J. A., and Wilkinson, G. S. (2017). Contrasting patterns of X-chromosome divergence underlie multiple sex-ratio polymorphisms in stalk-eyed flies. *Journal of Evolutionary Biology* **30**:1772–1784. doi: 10.1111/jeb.13140.

Presgraves, D. C., Severance, E., and Wilkinson, G. S. (1997). Sex chromosome meiotic drive in stalk-eyed flies. *Genetics* **147**:1169–1180.

R Core Team, (2016). R: A language and environment for statistical computing.

Reinhardt, J. A., Brand, C. L., Paczolt, K. A., Johns, P. M., Baker, R. H., and Wilkinson, G. S. (2014). Meiotic drive impacts expression and evolution of X-linked genes in stalk-eyed flies. *PLoS Genetics* **10**:e1004362. doi: 10.1371/journal.pgen.1004362.
