# Supplementary material for: Ejaculate sperm number compensation in stalk-eyed flies carrying a selfish meiotic drive element
Source: Heredity (Edinb). 2018 Nov 22;122(6):916–26. doi: 10.1038/s41437-018-0166-y (PMC6781104; doi:10.1038/s41437-018-0166-y)
Supplement: Supplementary file 2 — Supplementary Information B: Model tables and effect sizes [file 41437_2018_166_MOESM2_ESM.pdf]

Compensation of ejaculate sperm number in male stalk-eyed flies carrying a selfish meiotic drive element

Meade, L.C., Dinneen, D., Kad, R., Lynch, D.M., Fowler, K. & Pomiankowski, A.

Supplementary Information B: Model tables and effect sizes

Overview

Here we present the model tables and effect sizes for analyses reported in the main manuscript. All analyses were perfomed in R version 3.31 (R Core Team 2016). Generalised linear mixed effects models (GLMMs) used the *glmer* function from the package *lme4* (Bates et al. 2015) and general linear models (GLMs) used the *glm* function. Where count data were overdispersed, GLMs were fitted using a quasi- distribution and GLMMs utilised an observational level random effect (OLRE) (Harrison 2014). Spermathecae size and VR pouch count were normally distributed (Shapiro-Wilk test P > 0.1), and both were analysed with linear models using the *lm* function. P values were calculated using type II tests using the *Anova* function from the *car* package (Fox and Weisberg 2011). All reports of eyespan use residuals to avoid collinearity with male body size. Residual eyespan values are the residuals from a linear model after the variation in eyespan explained by thorax length has been removed (Dormann et al. 2013). Male ID is included as a random effect in analyses in sections SI-B1 and SI-B7 to account for repeated male measures.

Contents

SI-B1 Sperm allocation with female quality **3**

SI-B2 Sperm movement to the site of fertilisation (early period) **4**

SI-B3 Sperm movement to the site of fertilisation (late period) **6**

SI-B4 Sperm movement to the site of fertilisation across early and late periods **8**

SI-B5 VR pouch number **9**

SI-B6 Spermathecae size **9**

SI-B7 Sperm allocation across sequential matings **10**

SI-B8 Sperm allocation on a third mating **13**

SI-B1 Sperm allocation with female quality

SI-B1.1 Sperm presence in the spermathecae

```
glmer(sperm presence ~ male thorax + residual eyespan +
    female size * male type + (1|id), family = binomial)
```

```
Analysis of Deviance Table (Type II Wald chisquare tests)

Response: sperm_presence
                       Chisq Df Pr(>Chisq)
thorax                0.0670  1      0.796
residual_eyespan      0.0919  1      0.762
male_type             0.0390  1      0.843
female_size           0.8710  1      0.351
male_type:female_size 2.4800  1      0.116
```

```
                        Estimate Std. Error
(Intercept)                9.788     14.431
thorax                     1.415      5.467
residual_eyespan          -0.647      2.136
male_typeST               -5.788      4.801
female_sizeS              -5.323      4.511
male_typeST:female_sizeS   8.202      5.211
```

N = 109

SI-B1.2 Sperm number in the spermathecae

```
glmer(sperm number ~ male thorax + residual eyespan +
    female size * male type + (1|id) + (1 | OLRE), family = poisson)
```

```
Analysis of Deviance Table (Type II Wald chisquare tests)

Response: sperm_number
                      Chisq Df Pr(>Chisq)
thorax                1.184  1      0.276
residual_eyespan      1.615  1      0.204
female_size           0.470  1      0.493
male_type             0.267  1      0.605
female_size:male_type 0.071  1      0.789
```

```
                         Estimate Std. Error
(Intercept)                 2.448      1.511
thorax                      0.672      0.618
residual_eyespan            0.298      0.234
female_sizeS                0.227      0.379
male_typeST                -0.062      0.311
female_sizeS:male_typeST   -0.121      0.452
```

N = 97

SI-B2 Sperm movement to the site of fertilisation (early period)

SI-B2.1 Sperm presence in the VR

```
glm(VR sperm presence ~ male thorax + residual eyespan +
    female size * male type, family = binomial)
```

```
Analysis of Deviance Table (Type II tests)

Response: VR_sperm_presence
                      LR Chisq Df Pr(>Chisq)
thorax                   0.588  1      0.443
```

```
residual_eyespan         0.354  1      0.552
female_size              2.890  1      0.089 .
male_type                1.633  1      0.201
female_size:male_type    0.670  1      0.413
---
Signif. codes:  0 '***' 0.001 '**' 0.01 '*' 0.05 '.' 0.1 ' ' 1
```

```
                         Estimate Std. Error
(Intercept)                -2.240      2.700
thorax                      0.820      1.072
residual_eyespan           -0.251      0.423
female_sizeS               -1.040      0.706
male_typeST                 0.247      0.484
female_sizeS:male_typeST    0.639      0.790
```

N = 170

SI-B2.2 Sperm number in the VR

```
glm(filled pouch ~ male thorax + residual eyespan + female size * male type,
   family = quasipoisson)
```

```
Analysis of Deviance Table (Type II tests)

Response: filled_pouch
Error estimate based on Pearson residuals

                          SS Df     F Pr(>F)
thorax                 13.13  1 2.124  0.150
residual_eyespan        3.24  1 0.524  0.472
female_size             4.88  1 0.790  0.377
male_type               7.18  1 1.162  0.285
```

```
female_size:male_type   4.04  1 0.653  0.422
Residuals             420.25 68

                         Estimate Std. Error
(Intercept)                -1.580      2.229
thorax                      1.247      0.866
residual_eyespan            0.268      0.369
female_sizeS               -0.945      1.005
male_typeST                 0.239      0.389
female_sizeS:male_typeST    0.779      1.044
```

N = 74

SI-B3 Sperm movement to the site of fertilisation (late period)

SI-B3.1 Sperm presence in the VR

```r
glm(VR sperm presence ~ male thorax + residual eyespan +
    female size * male type, family = binomial)
```

```
Analysis of Deviance Table (Type II tests)

Response: VR_sperm_presence
                      LR Chisq Df Pr(>Chisq)
thorax                   0.012  1      0.913
residual_eyespan         0.754  1      0.385
female_size              6.442  1      0.011 *
male_type                0.483  1      0.487
female_size:male_type    0.343  1      0.558
---
Signif. codes:  0 '***' 0.001 '**' 0.01 '*' 0.05 '.' 0.1 ' ' 1
```

	Estimate	Std. Error
(Intercept)	0.282	3.220
thorax	0.145	1.323
residual_eyespan	-0.420	0.486
female_sizeS	0.619	0.564
male_typeST	0.110	0.407
female_sizeS:male_typeST	0.432	0.736

N = 213

SI-B3.2 Sperm number in the VR

```
glm(filled pouch ~ male thorax + residual eyespan + female size * male type,
   family = quasipoisson)
```

```
Analysis of Deviance Table (Type II tests)

Response: filled_pouch
Error estimate based on Pearson residuals
```

	SS	Df	F	Pr(>F)
thorax	0.85	1	0.273	0.602
residual_eyespan	2.23	1	0.721	0.397
female_size	0.08	1	0.025	0.874
male_type	2.71	1	0.874	0.351
female_size:male_type	3.27	1	1.056	0.306
Residuals	461.42	149		

	Estimate	Std. Error
(Intercept)	1.230	1.207
thorax	0.259	0.496
residual_eyespan	0.166	0.196

```
female_sizeS               -0.193      0.211
male_typeST                -0.235      0.169
female_sizeS:male_typeST    0.271      0.265
```

N = 155

SI-B4 Sperm movement to the site of fertilisation across early and late periods

```r
glm(VR sperm presence ~ male throax + residual eyespan + male type +
    time period, family = binomial)
```

```
Analysis of Deviance Table (Type II tests)

Response: VR_sperm_presence
                 LR Chisq Df Pr(>Chisq)
thorax              0.050  1      0.824
residual_eyespan    0.825  1      0.364
male_type           1.457  1      0.227
time_period        35.111  1     <2e-16 ***

                 Estimate Std. Error
(Intercept)        -0.927      1.980
thorax              0.178      0.801
residual_eyespan   -0.283      0.312
male_typeST         0.296      0.245
time_periodLate     1.278      0.221
```

N = 383

SI-B5 VR pouch number

```
lm(pouch number ~ female size)
```

```
Analysis of Variance Table

Response: pouch_number
             Df  Sum Sq Mean Sq F value    Pr(>F)
female_size   1  2640.3  2640.3  96.015 < 2.2e-16 ***
Residuals   450 12374.3    27.5

             Estimate Std. Error
(Intercept)    36.775      0.322
female_sizeS   -4.907      0.501
```

N = 452

SI-B6 Spermathecae size

SI-B6.1 Singlet

```
lm(singlet area ~ female size)
```

```
Analysis of Variance Table

Response: singlet_area
            Df  Sum Sq Mean Sq F value    Pr(>F)
female_size  1 3523241 3523241   46.56 < 2.2e-16 ***
Residuals   52 3934890   75671

             Estimate Std. Error
(Intercept)  2922.556     51.082
female_sizeS -512.270     75.075
```

N = 54

SI-B6.2 Doublet

```
lm(doublet area ~ female size)
```

```
Analysis of Variance Table

Response: doublet_area
            Df  Sum Sq Mean Sq F value    Pr(>F)
female_size  1 4420709 4420709   87.75 < 2.2e-16 ***
Residuals   62 3123478   50379

             Estimate Std. Error
(Intercept)  2756.398     36.900
female_sizeS -532.173     56.811
```

N = 64

SI-B7 Sperm allocation across sequential matings

SI-B7.1 Sperm presence in the spermathecea

```
glmer(sperm presence ~ male thorax + residual eyespan +
    mating order * male type + (1|id), family = binomial)
```

```
Analysis of Deviance Table (Type II Wald chisquare tests)

Response: sperm_presence
                        Chisq Df Pr(>Chisq)
thorax                 0.2900  1      0.590
residual_eyespan       0.4720  1      0.492
```

```
mating_order           1.8000  2      0.406
male_type              0.0132  1      0.908
mating_order:male_type 0.7210  2      0.697
```

```
                          Estimate Std. Error
(Intercept)                  6.570      6.952
thorax                      -1.548      2.874
residual_eyespan            -0.931      1.355
mating_order2                0.061      1.603
mating_order3               -0.311      1.646
male_typeST                 -0.623      1.485
mating_order2:male_typeST    0.814      1.790
mating_order3:male_typeST    1.590      1.876
```

N = 135

SI-B7.2 Sperm number in the spermathecae (across contiguous successful matings)

```r
glmer(sperm number ~ male thorax + residual eyespan +
    mating order * male type + (1|id) + (1 | OLRE), family = poisson)
```

```
Analysis of Deviance Table (Type II Wald chisquare tests)

Response: sperm_number
                       Chisq Df Pr(>Chisq)
thorax                 3.309  1      0.069 .
residual_eyespan       0.010  1      0.920
mating_order           0.198  2      0.906
male_type              1.372  1      0.241
mating_order:male_type 2.415  2      0.299
```

	Estimate	Std. Error
(Intercept)	2.760	1.046
thorax	0.799	0.439
residual_eyespan	0.019	0.192
mating_order2	0.495	0.322
mating_order3	0.230	0.350
male_typeST	0.056	0.264
mating_order2:male_typeST	-0.570	0.368
mating_order3:male_typeST	-0.224	0.395

N = 103

SI-B7.3 Sperm number in the spermathecae (across all successful matings)

```
glmer(sperm number ~ thorax + residual eyespan +
    mating order * male type + (1|id) + (1 | OLRE), family = poisson)
```

Analysis of Deviance Table (Type II Wald chisquare tests)

Response: sperm_number

	Chisq	Df	Pr(>Chisq)
thorax	1.605	1	0.205
residual_eyespan	0.002	1	0.966
mating_order	0.504	2	0.777
male_type	1.160	1	0.281
mating_order:male_type	2.848	2	0.241

	Estimate	Std. Error
(Intercept)	3.443	0.956
thorax	0.506	0.399
residual_eyespan	0.008	0.186
mating_order2	0.538	0.304

```
mating_order3                 0.234      0.319
male_typeST                   0.097      0.260
mating_order2:male_typeST    -0.576      0.348
mating_order3:male_typeST    -0.200      0.362
```

N = 118

SI-B8 Sperm allocation on a third mating

SI-B8.1 Sperm presence in the spermathecea

```
glm(sperm presence ~ male thorax + residual eyespan + male type,
   family = binomial)
```

```
Analysis of Deviance Table (Type II tests)

Response: sperm_presence
                  LR Chisq Df Pr(>Chisq)
thorax               2.060  1     0.1510
residual_eyespan     0.825  1     0.3640
male_type            2.910  1     0.0879 .

                  Estimate Std. Error
(Intercept)          4.449      2.867
thorax              -1.548      1.111
residual_eyespan     0.443      0.491
male_typeST          0.817      0.474
```

N = 111

SI-B8.2 Sperm number in the spermathecae

```
glm(sperm number ~ male thorax + residual eyespan * male type,
   family = quasipoisson)
```

```
Analysis of Deviance Table (Type II tests)

Response: sperm_number
                           LR Chisq Df Pr(>Chisq)
thorax                        0.795  1      0.373
residual_eyespan              2.751  1      0.097 .
male_type                     0.046  1      0.830
residual_eyespan:male_type    8.704  1      0.003 **

                             Estimate Std. Error
(Intercept)                     2.289      1.106
thorax                          0.378      0.427
residual_eyespan               -0.566      0.335
male_typeST                     0.069      0.224
residual_eyespan:male_typeST    1.179      0.397
```

N = 82

References

Bates, D., Mächler, M., Bolker, B., and Walker, S. (2015). Fitting linear mixed-effects models using *lme4*. *Journal of Statistical Software* **67**. doi: 10.18637/jss.v067.i01.

Dormann, C. F., Elith, J., Bacher, S., Buchmann, C., Carl, G., Carré, G., Marquéz, J. R. G., Gruber, B., Lafourcade, B., Leitão, P. J., Münkemüller, T., McClean, C., Osborne, P. E., Reineking, B., Schröder, B., Skidmore, A. K., Zurell, D., and Lautenbach, S. (2013). Collinearity: A review of methods to deal with it and a simulation study evaluating their performance. *Ecography* **36**:027–046. doi: 10.1111/j.1600-0587.2012.07348.x.

Fox, J. and Weisberg, S., (2011). An R companion to applied regression. Sage, Thousand Oaks, CA, 2nd edition.

Harrison, X. A. (2014). Using observation-level random effects to model overdispersion in count data in ecology and evolution. *PeerJ* **2**:e616. doi: 10.7717/peerj.616.

R Core Team, (2016). R: A language and environment for statistical computing.
